# Comparative Transcriptome Analyses of Deltamethrin-Resistant and -Susceptible *Anopheles gambiae* Mosquitoes from Kenya by RNA-Seq

**DOI:** 10.1371/journal.pone.0044607

**Published:** 2012-09-07

**Authors:** Mariangela Bonizzoni, Yaw Afrane, William Augustine Dunn, Francis K. Atieli, Goufa Zhou, Daibin Zhong, Jun Li, Andrew Githeko, Guiyun Yan

**Affiliations:** 1 Program in Public Health, University of California Irvine, Irvine, California, United States of America; 2 Centre for Global Health Research, Kenya Medical Research Institute, Kisumu, Kenya; 3 Department of Molecular Biology and Biochemistry, University of California Irvine, Irvine, California, United States of America; 4 Institute for Genomics and Bioinformatics, University of California Irvine, Irvine, California, United States of America; 5 Department of Chemistry and Biochemistry, University of Oklahoma, Norman, Oklahoma, United States of America; University of Kentucky, United States of America

## Abstract

Malaria causes more than 300 million clinical cases and 665,000 deaths each year, and the majority of the mortality and morbidity occurs in sub-Saharan Africa. Due to the lack of effective vaccines and wide-spread resistance to antimalarial drugs, mosquito control is the primary method of malaria prevention and control. Currently, malaria vector control relies on the use of insecticides, primarily pyrethroids. The extensive use of insecticides has imposed strong selection pressures for resistance in the mosquito populations. Consequently, resistance to pyrethroids in *Anopheles gambiae*, the main malaria vector in sub-Saharan Africa, has become a major obstacle for malaria control. A key element of resistance management is the identification of resistance mechanisms and subsequent development of reliable resistance monitoring tools. Field-derived *An. gambiae* from Western Kenya were phenotyped as deltamethrin-resistant or -susceptible by the standard WHO tube test, and their expression profile compared by RNA-seq. Based on the current annotation of the *An. gambiae* genome, a total of 1,093 transcripts were detected as significantly differentially accumulated between deltamethrin-resistant and -susceptible mosquitoes. These transcripts are distributed over the entire genome, with a large number mapping in QTLs previously linked to pyrethorid resistance, and correspond to heat-shock proteins, metabolic and transport functions, signal transduction activities, cytoskeleton and others. The detected differences in transcript accumulation levels between resistant and susceptible mosquitoes reflect transcripts directly or indirectly correlated with pyrethroid resistance. RNA-seq data also were used to perform a *de-novo* Cufflinks assembly of the *An. gambiae* genome.

## Introduction

The use of insecticides has been central to the fight against malaria, a disease that causes annually 665,000 deaths most in sub-Saharan Africa [1]. Pyrethroids (PYs) are the choice of insecticides for indoor-residual spray (IRS) and impregnating bednets because they meet the low toxicity and high efficacy requirements [2]. The extensive use of PYs imposes strong selection pressures on mosquito populations for increased resistance. Indeed, PYs resistance and cross-resistance between PYs and DDT have been detected in Africa [3]. As a consequence, insecticide resistance management is crucial for the success of malaria control, and subsequently, to promote economic development of malaria endemic countries. A key element of resistance management is the determination of resistance mechanisms in the malaria vectors [4].

The currently recognized PY resistance mechanisms in *Anopheles gambiae sensu stricto*, the primary malaria vector in sub-Saharan Africa, include a mutation in region II of the *para*-type sodium channel gene, that affects the affinity with which the insecticide binds and causes knockdown resistance (*kdr*) (target site resistance) [5],[6]. *Kdr* resistance is due to a single-base pair change that results in either a leucine-phenylalanine (L1014F) or a leucine-serine (L1014S) substitution at codon 1014 of the sodium channel gene [5], [6]. *Kdr* mutations are spatially widespread in Africa [3] and have reached high frequency levels. For example, the L1014F mutation is close to fixation in Ghana [7] and higher than 80% in Cameroon [8] and Burkina Faso [9], and the L1014S mutation has reached a frequency of more than 80% in Western Kenya [10]. The high *kdr* allele frequency likely results from wide-spread use of insecticide-treated bed nets (ITNs) [10], [11] and also from the intensive use of DDT and permethrin for control of cotton and rice pests in rural areas and for mosquito control in urban areas [12] because PYs used for agricultural purposes may leak into mosquito breeding sites and thus pose a selection pressure at the larval stage [11], [13]–[15]. However, the correlation between *kdr* allele frequency and PY resistance at the population level is low [10],[16],[17] and *kdr* allele frequency is not sufficient to predict the effectiveness of ITNs [18]. These observations support the conclusion that PY resistance involves mechanisms other than *kdr* mutations [17]. Other proposed insecticide resistance mechanisms includes overproduction of carboxyl-esterases, glutathione-S-transferases and P450-dependent monoxygenases that increase the rate of insecticide detoxification (metabolic resistance) [19]. Additionally, the thickening of cuticle and modification in the digestive tract lining may prevent or reduce insecticide penetration and absorption (penetration resistance) [3], [20],[21].

The understanding of the molecular mechanisms of insecticide resistance in *An. gambiae* has progressed rapidly with technological advancement. A number of techniques have been used in the research of insecticide resistance, including gene amplification-based techniques for the identification of *kdr* alleles [5], [6]; quantitative trait loci (QTL) analysis that have identified genetic loci linked to insecticide resistance [22]–[24]; and microarray techniques that study genome-wide expression profiling [20], [25]–[27]. However, microarrays are limited to the groups of genes spotted on the array and provide only relative expression levels. The recent development of RNA-seq technology is an improved method for gene-expression studies [28]. RNA-seq allows a holistic view of the transcriptome at a defined state, provides single-nucleotide level resolution and absolute rather than relative gene expression measurements. In mosquito, RNA-seq has been applied for *de novo* transcriptome assemblies in *An. funestus* [29] and *Ae. aegypti* [30], and expression profiles at specific life stages and tissues [31]–[34], but no attempt has been made to utilize this advanced technique to study the mechanism of resistance to insecticides in *Anopheles gambiae* mosquitoes.

We examined here the expression profile of deltamethrin-susceptible and resistant mosquitoes, as defined through the standard WHO tube bioassay [35]. Both resistant and susceptible mosquitoes derive from wild larvae with the same genetic background collected in Western Kenya. We also estimated the frequency of *kdr* mutation in *An. gambiae sensu latu* mosquitoes in association with insecticide usage in Western Kenya.

## Materials and Methods

### Study Sites and Phenotypic Resistance to Deltamethrin

**Figure 1:**
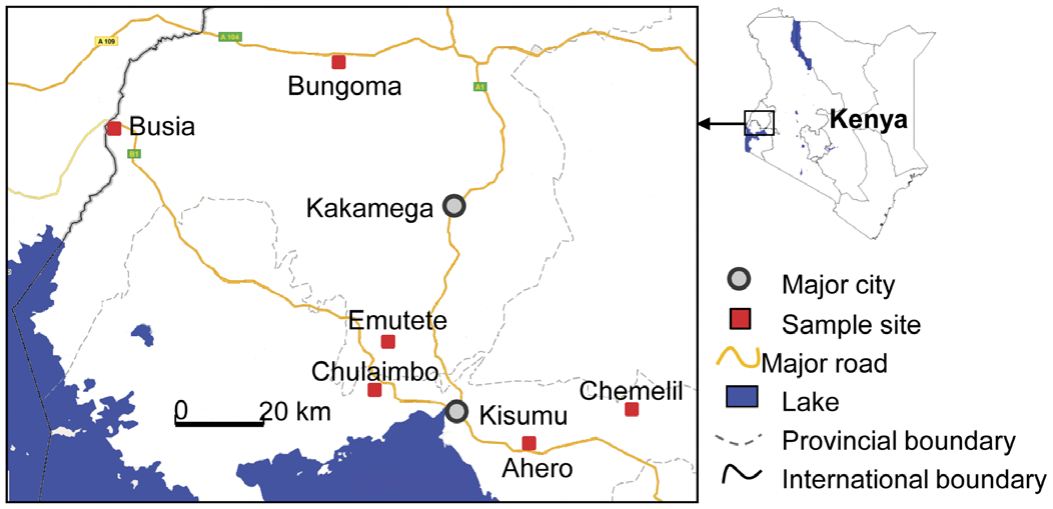
Map of Kenya showing the sampling sites. *Anopheles gambiae sensu lato* larvae were sampled in different breeding sites from Ahero, Chemelil, Emutete, Chulaimbo, Bungoma and Busia in Western Kenya.

Six populations were examined in this study: Ahero, Chemelil, Chulaimbo, Emutete, Bungoma and Busia (Figure 1, File S1). Within each location, *An. gambiae sensu latu* larvae were sampled from different breeding sites to avoid sampling larvae developing from eggs deposited from one mosquito and to capture the genetic variability of the population under analyses. Larvae were sampled between May and June 2010. The mosquitoes were reared to adulthood in the insectary of the Centre for Global Health Research, Kenya Medical Research Institute (KEMRI) in Kisumu, Western Kenya. During larvae sampling, a retrospective questionnaire survey was conducted of local inhabitants and field owners to determine insecticide usage (File S2). Deltamethrin is the principal insecticide used for vector control in Western Kenya. Phenotypic resistance to deltamethrin was tested by standard WHO tube bioassay [35] on *An. gambiae sensu latu* mosquitoes from the six locations. Between 115 and 661 mosquitoes per location were tested providing for biological replicates. The knockdown time was recorded for all tested mosquitoes at 10-minute intervals during the 60 minutes exposure time.

All mosquitoes alive and dead 24 hours after the end of the bioassay were preserved in 100% Ethanol or RNAlater® (Qiagen), respectively, for molecular analyses.

#### Ethics statement

No specific permissions were required for these locations. Locations where larvae sampling were conducted were not privately-owned or protected in any way. Larvae field collections did not involve endangered or protected species.

### Deltamethrin-susceptible and -resistant Mosquitoes for RNA-seq Analyses

Mosquitoes reared from larvae collected in November 2010 from Emutete were used for RNA-seq library preparation. We used Emutete population only because we obtained the highest number of resistant mosquitoes from this site. Bioassays for deltamthrin were conducted using one mosquito *per* tube test. The standard WHO bioassay for PY resistance requires exposing mosquitoes to papers impregnated with 0.05% deltamethrin for 60 minutes, followed by a resting period of 24 hours, after which dead (susceptible) and alive (resistant) mosquitoes are counted [35]. To study transcriptional changes between resistant and susceptible mosquitoes, a number of experimental procedures have been used to overcome post-mortem RNA degradation of susceptible mosquitoes. These include comparing bioassay-based resistant mosquitoes *versus* mosquitoes of a susceptible strain/population [36]–[38]; or mosquitoes of resistant *versus* susceptible strains [20],[25],[33] or first calculate the lethal time that kills 50% of the mosquitoes (LT_50_) and then subject the wild-caught mosquitoes to either the LT_50_ or not expose them to insecticide and assume as resistant the ones surviving the LT_50_ exposure and as susceptible the not exposed [26]. Here we defined resistant the mosquitoes alive 24 hours after the end of the bioassay and susceptible the mosquitoes knocked down early during the standard WHO tube bioassay [35]. This resistance classification criterion is reasonable because the insecticide dose used in the bioassay (0.05% deltamethrin) is a discriminating dose expected to kill 99% of the susceptible mosquitoes [35]. This experimental procedure allows sampling mosquitoes with the same genetic background, directly derived from field-collected larvae and subjected to the same laboratory conditions. Additionally, the percentage of early knock down mosquitoes was not significantly different between mosquitoes of the PY-susceptible Kisumu strain [39] and mosquitoes from Emutete-collected larvae (Figure S1).

Both susceptible and resistant mosquitoes were preserved in RNAlater® (Qiagen) and sent to UC Irvine.

### Mosquito Species Identification and *kdr* Genotyping

*Anopheles gambiae sensu latu* mosquitoes used in the bioassays could comprise mosquitoes of two morphologically indistinguishable species, co-occurring in our sampled localities: *An. gambiae sensu strictu* (here after referred as *An. gambiae*) and *Anopheles arabiensis* (File S1). An established molecular procedure [40] was used to discriminate between *An. gambiae* and *An. arabiensis*. Specifically, species identification was done by the Fast Tissue-to-PCR kit (Fermentas) using species-specific primers [40] and one leg of each stored mosquito. The *kdr* allele status was determined by direct sequencing of the fragment obtained by the polymerase chain reaction (PCR) with primers Adg1 and Adg2 [5],[6] or by the TaqMan assay following an established protocol [41]–[42].

### RNA-seq Library Preparation and Sequencing

For RNA-seq library preparation we used adult mosquitoes reared from larvae from Emutete and classified as deltamethrin-resistant or -susceptible as explained above. Additionally, we confirmed species identity of each mosquito before RNA extraction. Total RNA was extracted from *An. gambiae* mosquitoes using Trizol (Invitrogen).

After checking for the quality of RNA with an Agilent 2100 bioanalyzer, samples of nine resistant and nine susceptible mosquitoes were pooled and one RNA-seq library was prepared from each pool. Paired-end Illumina libraries were prepared and run for 80 cycles each end by the Expression Analysis Core at the UC-Davis Genome Center using standard Illumina procedures. Libraries were run at a concentration of 4–5 pM. RNA-seq data were deposited to the NCBI Gene Expression Omnibus (GEO) under accession number GSE39269.

### Data Analyses

#### Resistance bioassay data analysis

WHO guidelines were followed to analyze the results of the bioassays [35]. Specifically, mortality was scored 24 hours after exposure and the Abbot’s formula was used to correct for control mortality when required. A population was classified as “resistant” if mortality was less than 80%, as “suspected resistant” if mortality ranged between 80–97% and as “susceptible” if mortality was higher than 98% [35]. Fifty and 95% knock-down times (KT50 and KT95) were estimated by the logistic regression using the software STATISTICA (StatSoft, Tulsa, OK). The paired *t*-test was used to compare the mortality of female and male mosquitoes and to test for association between genotypes and phenotypes.

#### RNA-seq data analyses

TopHat [43] was used to map Illumina reads to the *An. gambiae* genome (version AgamP3). Cufflinks [44] was used to quantify transcript abundance in terms of Fragment Per Kilobase of exon model per Million mapped fragment (FPKM). Differential accumulation of transcripts between deltemethrin-resistant (R) and –susceptible (S) mosquitoes was tested by the Cuffdiff program within Cufflinks. Cuffdiff was run requiring a minimum alignment count of 10 and the upper-quartile-norm option, which can improve robustness of differential accumulation calls for less abundant transcripts. The Cuflinks suite was run twice. The first run used the published annotation of the *An. gambiae* genome as a strict reference guide for transcript annotation. Results from this run were used to identify significant differentially accumulation of transcripts between R and S mosquitoes. In the second run, the *An. gambiae* genome annotation was used as a guide of transcript annotation. Under this option, Cuflinks also looks for read-coverage supporting novel transcriptional events (NTE). Enrichment of Gene Ontology (GO) terms among differentially accumulated transcripts was tested by g:Profiler [45].

### Validation of RNA-seq Data

A total of 11 transcripts identified by RNA-seq to be accumulated differentially were chosen to perform quantitative real-time Polymerase Chain Reaction (qRT-PCR) (Table S1). Two novel transcriptional events (NTE) also were chosen for Reverse Transcription PCR (RT-PCR) (Table S1). Total RNA was extracted by TRIZOL (Invitrogen) from confirmed*An. gambiae* mosquitoes phenotyped as deltamethrin -resistant or –susceptible as described above and reared from two new independent larvae collections from Emutete and Bungoma. Following DNAse I (Promega) treatment, 1 µg of RNA was used for cDNA synthesis with superscript III (Invitrogen) and random primers. Quantitative real-time PCR reactions of 20 µl were performed in triplicate with SYBR Green Supermix (Biorad) and 0.3 µM of each primer on at least 9 S and 9 R mosquitoes from each location. Quantitative real-time PCR reactions were run on a Bio-Rad CFX thermal-cycler. No primer dimer was detected when inspecting the melting curves and primer pairs were chosen that displayed amplification efficiency between 80–105% [46]. Fold-changes in gene expression between 1) resistant mosquitoes from Emutete and susceptible mosquitoes from Emutete and 2) resistant mosquitoes from Bungoma and susceptible mosquitoes from Bungoma were derived by the comparative C_T_ method [47] using the S7 ribosomal protein gene as internal control [48]. Specifically, fold-changes between resistant and susceptible were derived by the formula: fold changes = 2^−ΔCT^Resistant/2^−ΔCT^Susceptible, with ΔC_T = _C_T_ gene of interest-C_T_ internal control [48].

Reverse transcription PCR on the same cDNA prepared for qRT-PCR was used for validation of two NTEs (Table S1).

## Results

### Susceptibility to Deltamethrin and *kdr* Genotype

PYs, primarily deltamethrin, are the main insecticides used in the study areas (File S2). A total of 1,605 wild mosquitoes were tested for phenotypic resistance to deltamethrin. Bioassay results show resistance in Bungoma and Busia (mortality<80%) and suspected resistance in Emutete (mosquito mortality was between 80–86%) (Table S2). Species identification between the morphologically indistinguishable *An. gambiae* and *Anopheles arabiensis* showed the exclusive presence of *An. arabiensis* in Ahero and Chemelil, a mixture of the two species in Bungoma, Busia and Chulaimbo and the presence of only *An. gambiae* in Emutete (Table S3). The latter was selected for RNA-seq material collection. The frequency of the *kdr* L1014S mutation in *An. gambiae* was over 94% in all tested sites and the predominant *kdr* genotype was homozygosity for the serine mutation (Table 1). Both serine and phenilaline substitutions were detected at codon 1014 among the *An. arabiensis* mosquitoes tested (Table S3).

**Table 1:** *Anopheles gambiae* and *An. arabiensis* deltamethrin resistance bioassay results.

Population*	No. mosquitoes tested	Percentage ofmortality ± standarderror**	Resistanceclassification***	*kdr* allelefrequency	Predominant *kdr* genotype (frequency)
Busia	130	66.5±13.7	Resistant	94.5	L1014S homozygote (89.1%)
Bungoma	661	78.7±5.6	Resistant	94.3	L1014S homozygote (89.5%)
Emutete	253	86.0±3.2	Probable Resistant	95.3	L1014S homozygote (90.6%)
Chulaimbo	315	97.6±2.4	Susceptible	95.2	L1014S homozygote (90.9%)
Ahero	131	96.6±8.7	Susceptible	7.5	L1014S heterozygote (73.4%)
Chemelil	115	92.4±9.3	Susceptible	86.5	L1014S heterozygote (69.2%)

* Species identification between *An. gambiae* and *An. arabiensis*, which was done after the bioassay, revealed the exclusive presence of *An. arabiensis* in Ahero and Chemilil. For Busia, Bungoma and Emutete, *kdr* allele frequency refers only to *An. gambiae* samples.

** Percentage of mortality refers to the percentage of mosquitoes that died 24 hrs after recovery from a 60 min exposure to the insecticides.

*** Resistance classification is based on WHO criteria: “Resistant” (mortality less than 80%), “Probable Resistant” (mortality rate ranged from 80–97%), and “Susceptible” (mortality more than 98%).

### RNA-seq Analyses of Resistance to Detamethrin

Two paired-end RNA-seq libraries were generated for pools of each R and S mosquitoes after having verified the *An. gambiae* identity of each mosquito and their *kdr* genotype. RNA-seq libraries were sequenced for 80 bp on each side, giving 64,900,398 and 73,785,834 reads for the resistant and susceptible sample, respectively.

**Figure 2:**
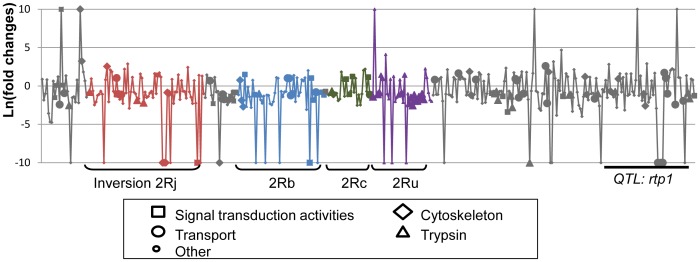
Distribution of differentially accumulated transcripts on chromosome 2R. Fold-changes in transcript accumulation levels between deltamethrin-resistant and susceptible mosquitoes for the significantly differentially accumulated transcripts mapping on chromosome 2R are presented. Various chromosomal inversions (2Rj, 2Rb, 2Rc and 2Ru) are indicated. The position of the QTL rtp1 is also indicated. For representation purposes, transcripts showing read coverage exclusively in susceptible or resistant mosquitoes were attributed an arbitrary fold-change of −10 or 10, respectively.

Transcript accumulation levels in a RNA-seq experiment are expected to reflect gene transcription, RNA stability and turn-over rate. When the AgamP3 version of the *An. gambiae* genome annotation was used as a strict reference guide for transcript annotation, a total of 13,229 transcripts, corresponding to 11,679 genes, had coverage in at least one sample and 1,093 transcripts were detected as significantly differentially accumulated (DA) between R and S mosquitoes (Table S4). RNA-seq read coverage for 19 and 53 transcripts was exclusive to the R and S samples, respectively (Table 2). A significant enrichment of differentially accumulated transcripts mapping to the right arm of chromosome 2 was observed (Table S5, Figure 2), including 6 and 27 transcripts with read coverage only in the R or S samples, respectively. Differentially accumulated transcripts mapping to 2R were associated mainly with trypsin (30 transcripts), cytoskeleton (25 transcripts), signal transduction (33 transcripts),transport (46 transcripts), and voltage-gated calcium channel (6 transcripts). Among these transcripts, a significant enrichment for GO terms associated with metabolic processes and proteolysis was observed (Figure 2). The 274 differentially accumulated transcripts mapping to 2L were enriched in GO terms associated with responses to abiotic stimuli, ion transport and metabolisms. The total of 299 transcripts mapping to 3R and 3L were enriched in GO terms linked to peptidase regulatory activity and transport. No enrichment of GO terms was observed for the 94 differentially accumulated transcripts mapping to the X chromosome.

**Table 2:** A summary of 1,093 transcripts differentially accumulated between deltamethrin-resistant and -susceptible mosquitoes based on fold-change differences.

	Fold changes^1^							
	>100	>50	>20	>10	>5	>2	<2	Total
Resistant > Susceptible^2^	1	1	5	26	59	219	18	329
Resistant < Susceptible^3^	2	4	20	48	129	446	43	692
Only in Resistant^4^								19
Only in Susceptible^5^								53

1 Significantly differentially accumulated transcripts were classified into 7 classes according to the amplitude of fold-changes.

2 Number of transcripts accumulated more in the resistant than in the susceptible sample.

3 Number of transcripts accumulated less in the resistant than in the susceptible sample.

4 Number of transcripts with exclusive read coverage in resistant mosquitoes.

5 Number of transcripts with exclusive read coverage in the susceptible mosquitoes.

### Association of Significantly Differentially Accumulated Transcripts with Functions Linked Previously to PY Resistance

**Figure 3:**
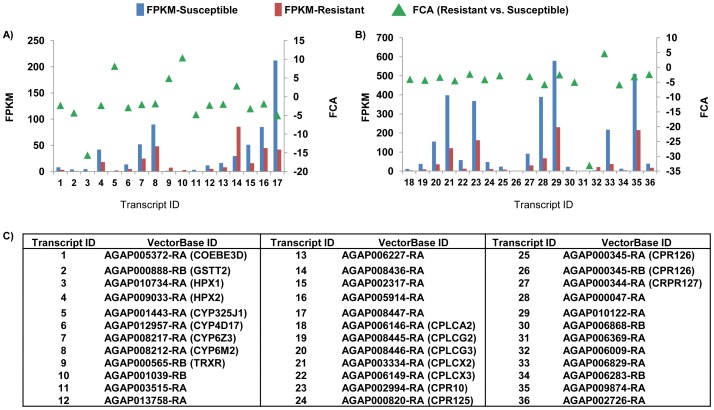
Significantly differentially accumulated transcripts associated to metabolic detoxification activities and coding for cuticular proteins. Absolute quantification level (in FPKM) and fold-changes in transcript accumulation levels (FCA) between deltamethrin-resistant and -susceptible mosquitoes are presented. **A**: transcripts related to metabolic detoxification activities; and **B**: transcripts coding for cuticular proteins; **C**: List of VectorBaseID.

A total of 17 transcripts included previously in the *An. gambiae* detoxification chip [25] were found significantly differentially accumulated (Figure 3). Transcripts AGAP001443-RA (CYP325J1), AGAP000565-RB (TRXR), AGAP001039-RB (cytochrome P450) and AGAP008436-RA (ABC transporter) were accumulated at higher levels in R than in S mosquitoes, with fold-changes between 2.88 (AGAP008436-RA) and 10.39 (AGAP001039-RB). All the other detox-related transcripts were accumulated less in R than in S mosquitoes.

A total of 110 transcripts coding for cuticular proteins showed read coverage in at least one sample, with 19 being significantly differentially accumulated between R and S mosquitoes. Only transcript AGAP006009-RA (CRP30) was accumulated at higher levels in R than in S mosquitoes. Transcript AGAP000345-RB (CRP126) had RNA-seq coverage only in S (FPKM = 2.32).

### Additional Transcripts Accumulated Differentially between R and S Mosquitoes

**Figure 4:**
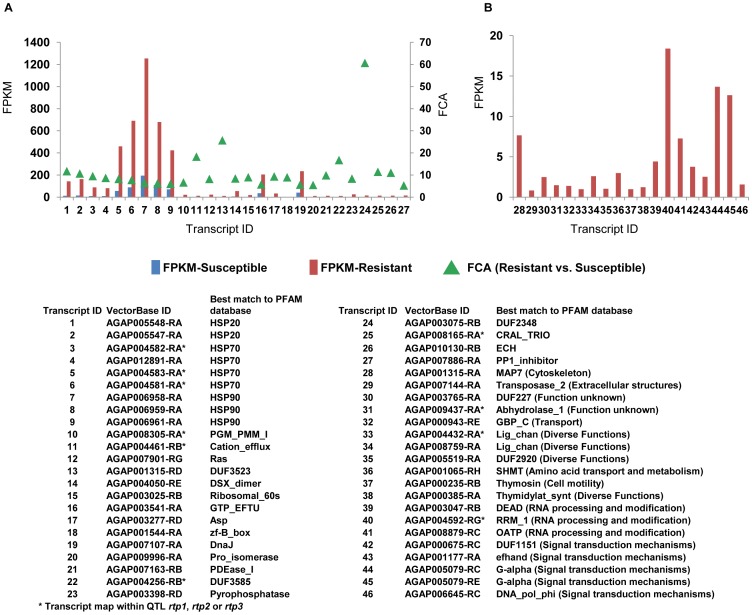
Transcripts with significantly higher accumulation in deltamethrin-resistant mosquitoes than deltamethrin-susceptible mosquitoes or with reads coverage only in deltamethrin-resistant mosquitoes. **A**: Transcripts expressed moderately in R mosquitoes (FPMK ≥10) and accumulated ≥5 folds in deltamethrin-resistant mosquitoes in reference to deltamethrin-susceptible mosquitoes; **B**: Transcripts with read coverage exclusively in the deltamethrin-resistant mosquitoes; and **C**: list of VectorBase idand best match to PFAM database of the transcrips.

The probe-independent RNA-seq approach identified other classes of transcripts significantly differentially accumulated between R and S mosquitoes. In particular, 27 transcripts were moderately expressed in R (FPKM ≥10) and ≥5 fold more accumulated in R than in S mosquitoes. Among these, five (AGAP004582-RA, AGAP004583-RA, AGAP004581-RA [hsp70], AGAP004461-RB [inorganic ion transport activity] and AGAP004256-RB [signal transduction mechanism]) and two (AGAP008305-RA [carbohydrate metabolism] and AGAP008165-RA [lipid metabolism]) transcripts mapped within QTLs are previously linked to resistance to permethrin [22], such as *rtp1* and *rtp2*, respectively (Figure 4A). Additionally AGAP004461-RB and AGAP004256-RB are among the transcripts with the highest fold-change in accumulation between R and S (18.3 and 16.7 respectively). Nineteen transcripts had read coverage only in the R sample, with FPKM values from 0.8 (AGAP007144-RA [extracellular structures]) to 18.4 (AGAP004592-RG [RNA processing and modification]). These transcripts were associated mainly with signal transduction mechanisms and three of them map within QTLs associated with permethrin resistance [22]. Transcripts AGAP004432-RA [inorganic ion transport and metabolisms] and AGAP004592-RG map within *rp1* and AGAP009437-RA [unknown function] maps within *rtp3* (Figure 4B). In general, among the differentially accumulated transcripts that have accumulated more in R than in S mosquitoes, there was a significant enrichment of GO terms associated with response to stress, heat and abiotic stimuli, transporter activity, specifically gated channel activity and ion transmembrane transporter activity, and binding functions.

A total of 109 transcripts were ≥5 fold accumulated in S than in R mosquitoes, with FPKM in S ≥10. No heat shock proteins were present in this group and there was an enrichment of transcripts associated with proteolysis functions. There was no enrichment for transcripts linked to response to stress. Seven transcripts mapped within *rtp1* and were associated with inorganic ion transport (AAEL004461-RA), cytoskeleton (AGAP004458-RC), RNA processing and modification (AGAP004592-RF and AGAP004549-RC) or diverse functions (AGAP004324-RA [putative translation initiation factor 3], AGAP004362-RA [metabolism], AGAP013117-RA [trypsin]).

### Correlation between RNA-seq and q-PCR Data

Eleven transcripts detected as differentially accumulated between S and R mosquitoes by RNA-seq were selected to perform qRT-PCR. Quantitative real-time data were generated for wild-mosquitoes from two field locations, Emutete and Bungoma. Mosquitoes were collected as larvae, raised to adults and phenotyped as deltamethrin-resistant or –susceptible.

**Figure 5:**
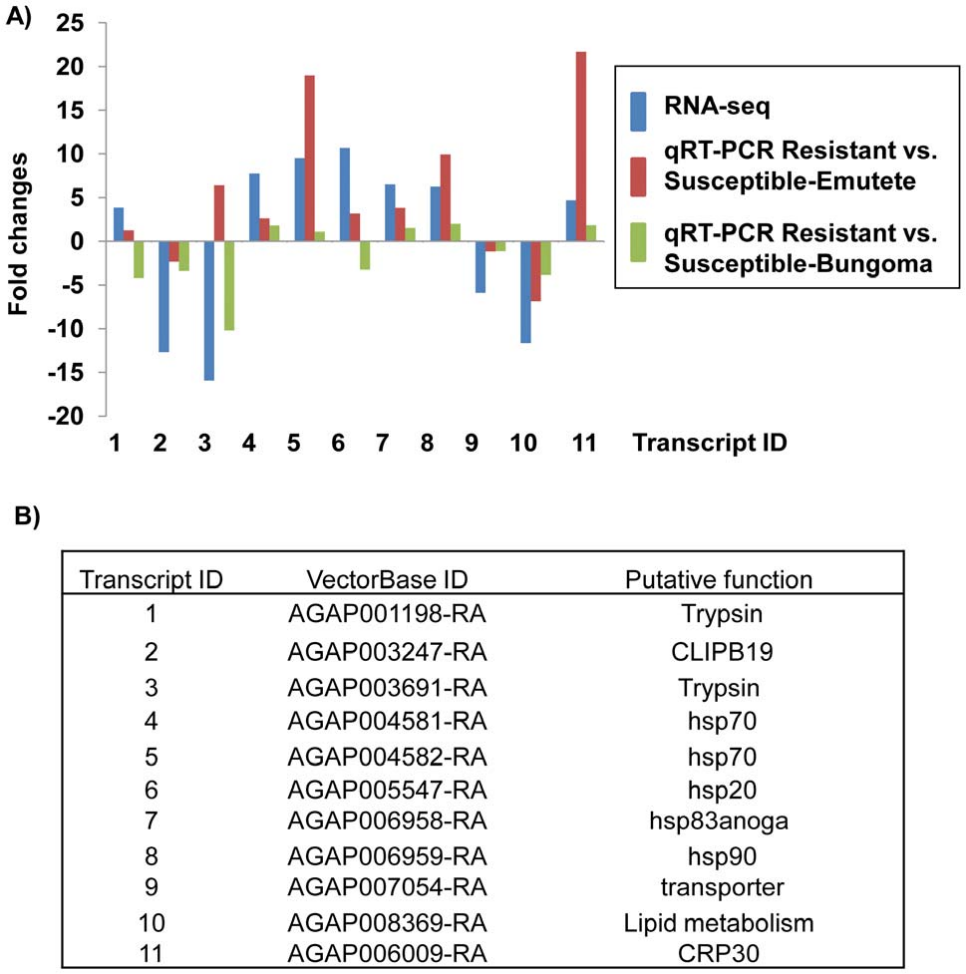
Comparison of RNA-seq and qRT-PCR in transcription determination of eleven selected genes. A: RNA-seq and qRT-PCR-based fold-changes in transcript accumulation levels. qRT-PCR fold-changes were derived comparing susceptible and resistant mosquitoes from Emutete (in red) and susceptible and resistant mosquitoes from Bungoma (in green); **B:** a list of VectorBase id and putative functions of the 11 transcripts examined.

When we compared RNA-seq data with qRT-PCR data generated from resistant-Emutete and susceptible-Emutete mosquitoes, direction of fold changes were conserved for 10 of the tested 11 transcripts. When we compared RNA-seq data with qRT-PCR data of Bungoma-resistant and Bungoma-susceptible mosquitoes, direction of fold changes was maintained for 9 of the 11 tested transcripts (Figure 5).

### Detection of NTEs Associated with Deltamethrin Resistance

**Figure 6:**
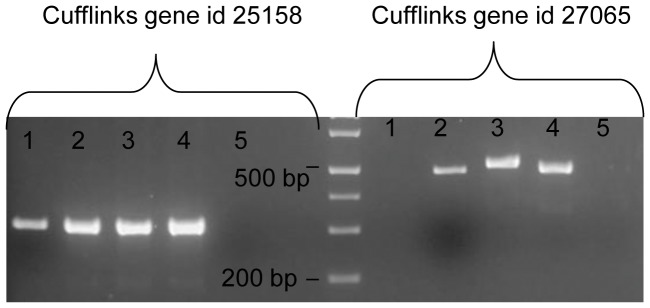
NTE validation. RT-PCR amplification on cDNA from mosquitoes from Emutete (lane 1) and Bungoma (lanes 2–4) of two NTEs identified when Cufflinks is run using the *An. gambiae* genome annotation as a simple guide of transcript annotation. Lane 5 is the negative control. Results for Cufflinks gene-id 27065 suggest the existence of isoforms.

A total of 8,057 new transcriptional features were identified when Cufflinks was run using the *An. gambiae* genome annotation as a guide rather than as a strict annotation (Table S6). Potentially novel isoforms and reads falling within currently annotated introns represent the majority of detected NTEs (89.6%). Approximately 16% of the NTEs documented ESTs aligned to their location. Further 982 NTEs have no documented ESTs aligning to within 1,000 bp +/− of their location and 431 have none within 10,000 bp +/−. The mapping positions and putative lengths suggest NTEs represent adjustments to the current *An. gambiae* genome annotation more frequently than novel genes. While complete annotation assessment and confirmation are beyond the scope of this study, transcription was confirmed for two events (Figure 6). Cufflinks gene-id 25158 maps within a region of the genome not linked yet to any chromosome (UNKN:30362579-30363863) and has no EST converge, suggesting that it is a novel open reading frame with a predicted length of 1284 bp. Cufflinks gene-id 27065 maps within chromosome X, overlapping genes AGAP000102 and AGAP000104.

## Discussion

Our comparative RNA-seq analyses of R and S mosquitoes indentified a total of 1093 transcripts significantly differentially accumulated. Differentially accumulated transcripts were distributed over the *An. gambiae* genome, with a significant enrichment on chromosome 2R, and several transcripts mapped within QTLs previously linked to permethrin resistance [22]. Deltamethrin and permethrin are both PY, but differ structurally in the presence of α substituted cyano (C≡N) group relative to the alcohol region of the ester functionality [49]. This structural difference does not affect their main target, the *para*-type sodium channel gene [50], but does alter the modality of interaction with the sodium channel with permethrin holding the channel open for a relatively shorter time than deltamethrin [51]. As a consequence, the finding of 17 differentially accumulated transcripts mapped within QTLs associated with permethrin resistance [22] is relevant to deltamethrin resistance even if compound-related variations in binding, effect and/or intensity are expected.

### Differentially Accumulated Transcripts Associated with Metabolic Detoxification or Coding for Cuticular Proteins

Studies on the metabolic pathways of PY degradation showed that initial steps of PY catabolism are similar in mammals, birds and insects and comprise oxidative, reductive and hydrolytic processes, involving esterases, P450 mono-oxigenases and, secondarily, glutation-S transferases [50]. On this basis, metabolic detoxification of PY compounds before they reach their target site has been recognized as an important resistance mechanism [50]. Three transcripts AGAP010737 (HPX1), AGAP008212 (CYP6M2) and AGAP008217 (CYP6Z3) have been consistently identified by microarray analyses as over-expressed in PY-resistant *An. gambiae* strains or wild populations from West Africa in comparison to the Kisumu strain or field susceptible mosquitoes [20], [31], [36], [37]. Moreover, direct evidence that the cytochrome P450 6M2 metabolizes deltamethrin was provided recently [52]. In our comparisons, CYP6M2, CYP6Z3 and HPX1 encoding-transcripts were accumulated less in R than in S mosquitoes (Figure 2A). Contrasting differential accumulation of redox-associated transcripts in comparisons of PY-resistant/susceptible mosquitoes has been observed and probably reflect biological differences among strains or populations of various geographic origins [20], [31], [36], [37]. For instance, in comparison with the PY-susceptible Kisumu strain [39], the glutathione-S transferase gene GSTE2 (AGAP009194) was over-expressed in the permethrin tolerant RSP and in the DDT-resistant ZAN/U strains from West Africa [25], but under-expressed in the permethrin resistant Odumasy strain from Ghana [20]. The thioredoxine gene TPX2 (AGAP011054) was expressed at higher levels in the PY-resistant Ojoo than in the PY-susceptible Orogun population [36], but expressed at lower level in the PY-resistant Ipokia than in the PY-susceptible Alakia population from south and south-central Nigeria [37]. Finally, the P-450 mono-oxygenase CYP6AG2 (AGAP013511) was over-expressed in Ojoo strain in comparison to Orogun strain [36], but under-expressed in the RSP with respect to the Kisumu strains [25].

Our data show that detox-transcripts tended to be accumulated at lower levels in R than in S mosquitoes and the transcript encoding for NADH cytochrome b_5_ reductase (AGAP011300-RA), which is required for P450 activity, was not differentially accumulated between R and S mosquitoes (Table S4). This result is consistent with limited reports of metabolic detoxification mechanisms in *An. gambiae* mosquitoes from Western Kenya [42]. Interestingly, data on the main Asian malaria mosquito *Anopheles sinensis* support the hypothesis that mosquitoes from different genetic background have evolved different mechanisms of resistance to PYs, with either a preponderance of metabolic detoxification mechanisms or mutations in the target site [53]. Deltamethrin acts as a competitive inhibitor of esterase activity and is removed by binding rather than being catabolized through hydrolyses in the peach-potato aphids *Myzus persicae* [50]. Hence, it would be interesting to test if mosquitoes from West and East Africa show alternative mutations in esterase-coding genes that could affect insecticide-binding. This could be part of the reasons for the differences in metabolic detoxification observed in mosquitoes from West and East Africa.

An additional mechanism of insecticide resistance may result from cuticular proteins that limit insecticide penetration or absorption [50]. Decreased insecticide penetration is considered an additive resistance factor, with limited effect on its own, found only in association with either enhanced metabolism or target-site mutations [50]. The transcript encoding for the cuticular protein CRP30 was the only one with increased accumulation in R. This was confirmed by qRT-PCR data. While this result suggests that CRP30 merits further investigation, the estimate of accumulation levels for other cuticular proteins may be under-represented at the whole body level as cuticular proteins are thought to be synthesized only by epidermal cells [54]. CRP30 belongs to the RR-1 group of the proteins with the chitin binding motif defined by the R&R Consensus [55] and is associated with soft (flexible) cuticle [56]. Soft, more hydrated, cuticle is important when movement is required, such as during the larval and adult stages, and is present in regions forming joints or connecting rigid segments [54]. Detailed analyses of developmental expression pattern of CRP30 showed transcript presence throughout the larval stages with peak abundance for 24h pupae and minimal transcript detection in sugar-fed adult mosquitoes [57]. Furthermore, this transcript is responsive to the circadian cycle [58] and shows a maximum accumulation 3 hours post blood-meal [59], indicating that its expression can be modulated in adult female mosquitoes. It is important to investigate the tissue-specific expression of CRP30 and its relative location and abundance with respect to other cuticular proteins expressed in adult mosquitoes [57] in order to clarify its putative role in insecticide resistance.

### Additional Differentially Accumulated Transcripts Associated with PY Resistance

Whole-genome expression profiling by RNA-seq showed differential accumulation, between R and S mosquitoes, of several additional classes of transcripts, including transcripts encoding for heat shock proteins or associated with proteolysis, ion transport, signal transduction, metabolic activities and cytoskeleton.

#### Transcripts encoding for heat shock proteins

All differentially accumulated transcripts encoding for heat shock proteins were found accumulated more in R than in S mosquitoes. Real-time quantitative PCR data confirmed higher accumulation of four heat shock protein-encoding transcripts in R versus S mosquitoes from Emutete and also from Bungoma. Pyrethroids are known to induce rapid insect knock-down, but they are poor in killing mosquitoes [50]. A consequence of this “slow-killing” action is a general physiological stress in the mosquito body. Heat shock proteins are key components of response to environmental stress [60] and in mosquitoes they have been associated with dehydratation tolerance [61], protection from high temperature blood meal [62], defense from pathogens [63] and a short-term response to exposure to sub-lethal doses of permethrin [64]. The resistant mosquitoes in this study were sampled 24 hours after deltamethrin exposure; hence the higher accumulation of heat shock protein-encoding transcripts detected in R mosquitoes may reflect long-term effects of insecticide exposure and may be part of a general stress response. Our results are also compatible with the hypothesis that R mosquitoes have a higher constitutive level of heat shock proteins that would confer an immediate advantage with respect to S mosquitoes upon insecticide exposure.

#### Transcripts encoding for proteins with a proteolysis activity

Transcripts associated with proteolysis have already been linked to insecticide resistance, with the hypothesis that a balance between protein degradation and synthesis is required to meet energy demands during stress [65]. An over-expression of transcripts linked to peptidase activity was detected in DDT-resistant *Drosophila melanogaster* [65] and in the *An. gambiae* RSP and ZAN/U strains [64] and a higher proteolysis activity was observed in DDT-resistant *versus* -susceptible *Musca domestica* strain [66]. RNA-seq data show that four (AGAP004700-RN, AGAP010243-RA, AGAP008290-RA, AGAP001198-RA) and eight (AGAP010062-RA, AGAP005108-RB, AGAP001023-RC, AGAP006904-RB, AGAP005881-RA, AGAP012945-RA, AGAP004920-RA, AGAP011952-RA) transcripts with trypsin or peptidase domains, respectively are accumulated more in R than in S.

#### Other casses of differentially accumulated transcripts

Transcripts associated with metabolic functions**,** transport activities and cytoskeleton were enriched among significantly differentially accumulated transcripts, suggesting a link between these physiological processes and phenotypic resistance. PYs are lipophilic compounds shown to localize within the hydrocarbon core of model membrane systems formed of phospholipids and appear to have a disordering effect on the interior regions of the bilayer [67]. Such localization may influence the PY availability to the *para*-sodium channel, which is a transmembrane protein [5]. PYs action on the sodium channel destabilizes the equilibrium of Na^+^ and K^+^ ions across the membrane, which immediately affects the membrane potential and is the basis of PY toxicity [50]. PYs induce repetitive channel firing, which results in a continuous influx of Na^+^ ions. This coupled with the PY disordering effect of membrane lipid packing may also disturb the overall insect metabolism, thus requiring the intervention of proteins with metabolic and transport functions and contributing to the biological actions of PYs. Alternatively, modifications in the metabolisms could be an effect of the stress induced upon insecticide exposure. A direct alteration of the activity of enzymes involved in carbohydrate and lipid metabolisms, such as succinate dehydrogenase and gluocose-6-phosphase dehydroganase, was observed in the common carp, *Cyprinus carpio*, following PY exposure [68]. Additionally, it was observed that microtubule disruption leads to down-regulation of several P450 dependent mono-oxygenases in mammals [69].

Several transcripts whose products are involved in signal transduction activity were differentially accumulated, indicative of a possible relationship with deltamethrin resistance. This effect may be indirect as was concluded in a study of *Culex quinquefasciatus*, where the knockdown of expression of a rhodopsin-like GPCR gene caused a decrease in the expression of several cytochrome P450 genes accompanied by a reduction of tolerance to permethrin [70].

In conclusion, the results obtained here are consistent with previous reports that the *kdr* genotype cannot fully predict PY resistance phenotype [17]. The RNA-seq analyses of R and S mosquitoes identified a number of differentially accumulated transcripts located in QTLs previously linked to resistance to PYs and/or associated with functions linked to PY resistance in other organisms or consistent with PY mode of action. In addition, the application of RNA-seq data to *de novo* Cufflinks assembly supports the need for continued amendment of the current *An. gambiae* genome annotation. Our knowledge of insecticide resistance would greatly benefit from a revision of the genome annotation as evidenced by several NTEs or novel isoforms detected as significantly differentially accumulated between R and S mosquitoes.

## Supporting Information

Figure S1 **Time course for percentage of adult** ***Anopheles gambiae*** **mosquitoes being knocked down during the WHO deltamethrin bioassay.** The percentage of knockdown mosquitoes was calculated over 9 replicates for mosquitoes from Emutete and 10 replicates for mosquitoes of the laboratory-reared susceptible reference Kisumu strain, with 20–50 mosquitoes per replicate. The vertical bar stands for standard deviation. The statistical significance in the mean knockdown time between the Emutete population and the Kisumu strain is shown, NS = Non-significant, * P<0.05, ** P<0.01 after correction for multiple comparisons.
(TIF)

Table S1 **List of primers used for qRT-PCR and RT-PCR.**
(XLSX)

Table S2 **Susceptibility to deltamethrin of wild** ***Anopheles gambiae*** **s.l. mosquitoes from the six study sites in Western Kenya as determined by the standard WHO tube test.**
(PDF)

Table S3 **Results of molecular identification of** ***Anopheles gambiae*** **and** ***An. arabiensis*** **and** ***kdr*** **genotype.**
(XLSX)

Table S4 **Cuffdiff output using the** ***Anopheles gambiae*** **genome annotation as a strict reference guide for transcript annotation.** Significantly differentially accumulated transcripts are in bold.
(XLSX)

Table S5 **P values associated with a hypergeometric test are shown for all significantly differentially accumulated transcripts (A), differentially accumulated transcripts higher accumulated in R versus S (B) or lower accumulated in R versus S (C ), transcripts with read coverage only in R (D) or S (E), respectively.**
(PDF)

Table S6 **List of novel transcriptional events significantly differentially accumulated detected when Cufflinks is run using the** ***An. gambiae*** **genome annotation as a guide for transcript annotation.**
(XLSX)

File S1 **Detailed information on the six localities in Western Kenya where mosquitoes were collected.**
(PDF)

File S2 **Survey on insecticide usage in six study sites in Western Kenya.**
(PDF)
