# Supplementary figures and images for: Comparative Transcriptome Analyses of Deltamethrin-Resistant and -Susceptible Anopheles gambiae Mosquitoes from Kenya by RNA-Seq
Source: PLoS One. 2012 Sep 7;7(9):e44607. doi: 10.1371/journal.pone.0044607 (PMC3436877; doi:10.1371/journal.pone.0044607)

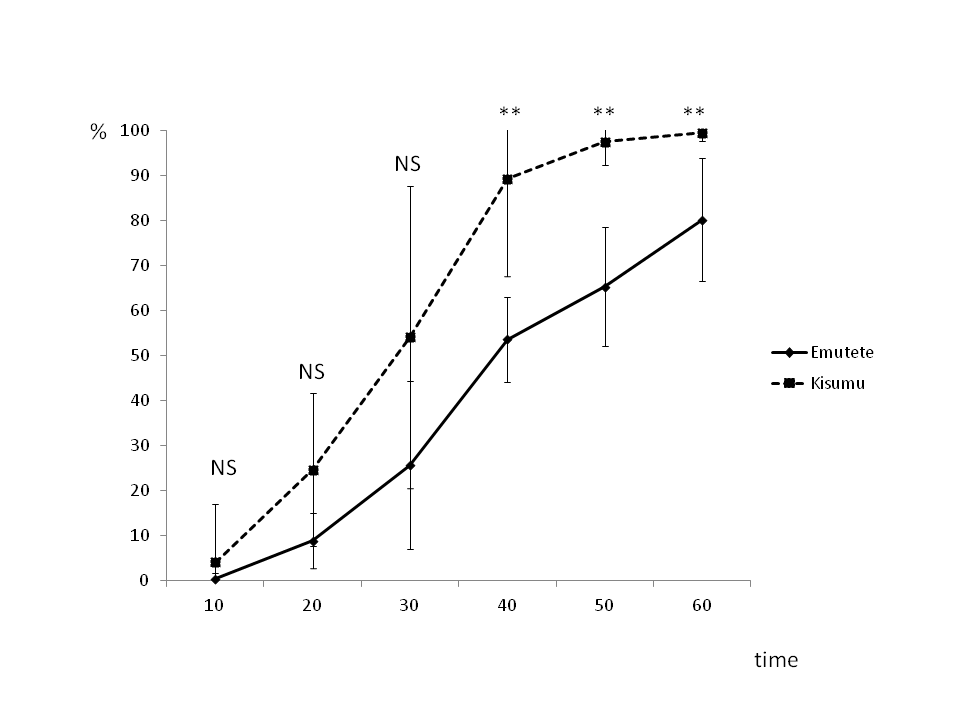

Supplement: Figure S1 — Time course for percentage of adult Anopheles gambiae mosquitoes being knocked down during the WHO deltamethrin bioassay. The percentage of knockdown mosquitoes was calculated over 9 replicates for mosquitoes from Emutete and 10 replicates for mosquitoes of the laboratory-reared susceptible reference Kisumu strain, with 20–50 mosquitoes per replicate. The vertical bar stands for standard deviation. The statistical significance in the mean knockdown time between the Emutete population and the Kisumu strain is shown, NS = Non-significant, * P<0.05, ** P<0.01 after correction for multiple comparisons. (TIF) [file pone.0044607.s001.tif]
