# Supplementary material for: Comparative Transcriptome Analyses of Deltamethrin-Resistant and -Susceptible Anopheles gambiae Mosquitoes from Kenya by RNA-Seq
Source: PLoS One. 2012 Sep 7;7(9):e44607. doi: 10.1371/journal.pone.0044607 (PMC3436877; doi:10.1371/journal.pone.0044607)
Supplement: Table S2 — Susceptibility to deltamethrin of wild Anopheles gambiae s.l. mosquitoes from the six study sites in Western Kenya as determined by the standard WHO tube test. (PDF) [file pone.0044607.s003.pdf]

TableS2. Susceptibility to deltamethrin of wild *Anopheles gambiae s.l.* mosquitoes from 6 sites in Western Kenya as determined by the standard WHO tube test.

**Study site**	**N. mosquitoes tested** total (females)	**% mortality ± 95% C.I.** total (females)	**KT50** min	**KT95** min
kisumu strain	258 (205)	100 (100)	27.39	42.8
Ahero	131 (62)	96.61 ± 8.71 (93.33 ± 17.14)	36.26	*
Chemelil	115 (69)	92.39 ± 9.33(90.49 ± 11.23)	32.25	54.97
Chulaimbo	315 (187)	97.64 ± 2.36 (97.04 ± 2.32)	40.57	*
Emutete	253 (133)	86.01 ± 3.24 (80.36 ± 5.46)	39.57	*
Bungoma	661 (337)	78.70 ± 5.60 (68.97 ± 7.86)	43.37	*
Busia	130 (77)	66.45 ± 13.73 (51.80 ± 17.32)	39.04	*

* the maximum percentage (± 95% CI) of mosquitoes that were knocked down after 60 minutes (± 8.70) of exposure was for Ahero 91.34, Chulaimbo 80.92 (± 10.91), Emutete 80.11 (± 10.51), Bungoma 80.75 (± 5.93), and Busia 89.81 (± 9.08)

** the maximum percentage (± 95% CI) of mosquitoes that were knocked down after 60 minutes (± 23.97) of exposure for Emutete was 86.52
