# Supplementary material for: Comparative Transcriptome Analyses of Deltamethrin-Resistant and -Susceptible Anopheles gambiae Mosquitoes from Kenya by RNA-Seq
Source: PLoS One. 2012 Sep 7;7(9):e44607. doi: 10.1371/journal.pone.0044607 (PMC3436877; doi:10.1371/journal.pone.0044607)
Supplement: Table S5 — P values associated with a hypergeometric test are shown for all significantly differentially accumulated transcripts (A), differentially accumulated transcripts higher accumulated in R versus S (B) or lower accumulated in R versus S (C ), transcripts with read coverage only in R (D) or S (E), respectively. (PDF) [file pone.0044607.s006.pdf]

Table S5.

P values associated with a hypergeometric test are shown for all significanlty differentially accumulated transcripts (A), transcripts accumulated more in resistant than in susceptible (B) or accumulated less in resistant than in susceptible (C) mosquitoes, transcripts with read coverage only in resistant (D) or suscpetible (E), respectively.

Cluster	A	B	C	D	E
X	0.57	1	0.999	1	1
2R	3.33E-08	1	0.999	1	1
2L	0.11	1	0.999	1	1
3R	0.996	1	1	1	1
3L	0.999	1	0.999	1	1
UNK	0.999	0.999	1	1	1
