# Supplementary material for: Comparative Transcriptome Analyses of Deltamethrin-Resistant and -Susceptible Anopheles gambiae Mosquitoes from Kenya by RNA-Seq
Source: PLoS One. 2012 Sep 7;7(9):e44607. doi: 10.1371/journal.pone.0044607 (PMC3436877; doi:10.1371/journal.pone.0044607)
Supplement: File S1 — Detailed information on the six localities in Western Kenya where mosquitoes were collected. (PDF) [file pone.0044607.s008.pdf]

File S1

Study sites and survey on insecticide usage

Anopheles gambiae larvae were sampled from more than 200 breeding sites from six locations in Western Kenya. Chulaimbo (00.03572°S, 034.62196°E, altitude 1328m) is in the district of Kisumu, Ahero (00.17259°S, 034.91983°E, altitude 1162m) and Chemelil (00.11086°S, 035.11155°E, altitude 1217) are in the Nyando district, all of which are in the Nyanza province. Emutete (00.02635°N, 034.63316°E, altitude 1561m), Bungoma (00.54057°N, 034.56410°E, altitude 1545m) and Busia (0.4530°N; 034.1250°E, altitude 1179m) are in the Western Province. Emutete belongs to the Emuhaya district, Busia to the Busia district and Bungoma to the Bungoma South district. Malaria transmission is perennial in the lowland sites of Ahero, Chemelil, Chulaimbo and Busia. A mixture of *An. gambiae sensu stricto* (s.s.) and *An. arabiensis* is expected in all lowland sites [1], except Ahero, where only *An. arabiensis* has been collected since the late '90 [2]. In Chulaimbo, the entomological inoculation rate (EIR) was 31.1 infectious bites/person/year in a 12 month period ending in 2004 [3]. The highland sites of Emutete and Bungoma show low seasonal malaria transmission, with peaks at the end of the long (early April to early June) and short (October-November) rainy seasons and high year-to-year variation. The prevalent malaria vector in the highlands is *An. gambiae* s.s. The EIR in Emutete was 11.98 infectious bites/person/year in 2009 [4]. Agricultural activity is particularly intense in Ahero, Chemelil and Bungoma. In Ahero the principal crop harvested is rice. In Chemelil and Bungoma sugar cane is the main crop cultivated and Bungoma site is the home of the largest sugar cane factory in Kenya. Additionally, there are numerous small-holder sugar mills, and maize, pearl millet and sorghum also are grown for subsistence. The economy in Busia is dependent mainly on fishing and trading with the neighboring Uganda, with small cultivations of maize, cassava, millet, sweet potatoes and beans. The Emuhaya district is characterized by large tea plantations, with smaller cultivation of maize, potatoes and bananas. Chulaimbo is a rural community with small cultivation of potatoes, bananas and maize. During larvae sampling, a quality survey was conducted on local inhabitants to document insecticide usage (Supplemental Table S1). Data were collected following a standardized questionnaire that included questions on crops harvested; insecticides used, including brand name, time and operation dosage for crop treatment and animal protection; ITN usage and IRS in interviewee houses. For each site, 20 forms were

collected and results are summarized in Additional Table 1. A mixture of multifamily compounds and single-family houses was present in all sites, with a prevalence of multifamily compounds in Ahero, Busia and Emutete. Apart from specialty crops such as rice, cultivated intensively in Ahero, and sugar cane, harvested in Chemelil and Bungoma, the other crops grown were similar in all localities: mainly maize, sweet potatoes, bananas and garden vegetables. The percentage of farmers using pesticides, the type of pesticides and the frequency of applications varied greatly among sites. In general, for agricultural purposes, organophosphate-based insecticides were more commonly used than compounds containing PY. The site with the highest usage of pesticides was Ahero, probably due to the increased in rice production in 2008 following the FAO Initiative on Soaring Food Prices (ISFP) and the implementation of the local irrigation scheme [5]. In all sites, the same compound, composed of amitraz (1.5% m/v), deltamethrin (0.50% m/v) and pipernyl butoxide (3 % m/v), was used to spray animals for tick protection, even if there was extreme variability in the frequency of applications, ranging from weekly to an application every six months. Bed nets coverage was over 90% in all sites, with a predominance of long-lasting bed nets. However, the number of bednets *per* household was clearly insufficient to provide coverage to all the inhabitants and when bed nets requiring re-treatment were present; in only one instance recommended guidelines were followed. IRS was provided only in Emutete, with ICON (lambda cyhalothrin) being the most used insecticides. The large variability encountered among the sites analyzed, both in terms of type of insecticides and frequency of usage, prevents a quantitative analyses of the data, however, the surveys support the conclusion that the main sources of insecticide in our sites are bednets and animal protection.

Phenotypic resistance

The pyrehroid compound deltamethrin is the mainstay for vector control in Western Kenya. Phenotypic resistance to deltamethrin was tested with the standard WHO cone assay [6]. Briefly, mosquitoes were reared to adults and 2-4 day-old non-bloodfed old adults were exposed to deltamethrin (0.05%) for 60 min. Batches of mosquitoes from the same site were used as control mosquitoes and were exposed to test paper impregnated with the insecticide carrier only (no insecticide). The quality of the insecticide impregnated papers was tested on mosquitoes of the susceptible-reference Kisumu strain [7]. Susceptibility tests were conducted at ambient

temperature and humidity (25-29°C and 70-80% relative humidity). Mosquito knockdown time and survivorship were recorded.

REFERNCES

1. Githeko AK, Service MW, Mbogo CM, Atieli FK (1996) Resting behavior, ecology and genetics of malaria vectors in large scale agricultural areas of Western Kenya. *Pasasitologia* 38: 481-489.

2. Stump AD, Atieli FK, Vulule JM, Besanski NJ (2004) Dynamics of the pyrethroids knockdown resistance allele in western Kenyan populations of *Anopheles gambiae* in response to insecticide-treated bed net trials. *Am J Trop Med Hyg*, 70: 591-596.

3. Ndenga B, Githeko A, Omukunda E, Munyekenye G, Atieli H, Wamai P, Mbogo C, Minakawa N, Zhou G, Yan G (2006) Population dynamics of malaria vectors in western Kenya highlands. *J Med Entomol*, 43: 200-206.

4. Fillinger U, Ndenga B, Githeko A, Lidsay SW (2009) Integrated malaria vector control with microbial larvicides and insecticide-treated nets in western Kenya: a controlled trial. *Bull World Health Organ* 2009, 87: 655-665.

5. FAO Initiative on Soaring Food Prices. Availabe at: http://www.fao.org/isfp/isfp-home/en/. Accessed May 04th, 2012

6. WHO (1998) Test procedures for insecticide resistance monitoring in malaria vectors, bio-efficacy and persistence of insecticides on treated surfaces. WHO/CDS/CPC/MALI/9812.

7. Vulule JM, Beach RF, Atieli FK, Roberts JM, Mount DL, Mwangi RW (1994) Reduced susceptibility of *Anopheles gambiae* to permethrin associated with the use of permethrin-impregnated bednets and curtains in Kenya. *Med Vet Entomol* 8:71-75.
