# Supplementary material for: Comparative Transcriptome Analyses of Deltamethrin-Resistant and -Susceptible Anopheles gambiae Mosquitoes from Kenya by RNA-Seq
Source: PLoS One. 2012 Sep 7;7(9):e44607. doi: 10.1371/journal.pone.0044607 (PMC3436877; doi:10.1371/journal.pone.0044607)
Supplement: File S2 — Survey on insecticide usage in six study sites in Western Kenya. (PDF) [file pone.0044607.s009.pdf]

File S2.

Survey on insecticide usage in six localities in Western Kenya.

Study site	House demography Adults/kids	Trade name-Insecticide class (dosage, % use)	Vector control for animals	IRS (Insecticide class, % houses treated)	Bednets % usage-Type of bed nets (%[1]), N. bednets/household
Ahero	5.1/5.85	Dursban-OP (depends on pest, 5%) Furadan 5G-OP (depends on pest, 10%) Tata Umeme-PY 2.5EC (15-20ml/20l, 55%),Ogor 40EC-OP (30-40ml/20l, 45%) Mocap GR10-OP (depends on pest, 45%)	Triatix-PY[2] (55%)	none	95%, ITN (78.95%); LLTN (89.47%), 3.42
Chemelil	2.15/2.9	Diazol-OP (40-50 ml/20l, 5%) Danadim-OP (400g/l, 5%)	Triatix-PY (65%)	Doom-PY (0.03% w/w, 10%)	95%, ITN (47.37%), LLTN (84.21%), 2.25
Chulaimbo	4.8/6.75	Diazol-OP (40-50 ml/20l, 10%) Danadim-OP (400g/l, 5%)	Triatix-PY (60%)	Not known, 5%	90%, ITN (33.3%), LLTN (88.9%), 3.22
Emutete	4/4.7	Diazol-OP (40-50 ml/20l, 15%) Pyrinex 48EC-OP (400g/l, 5%)	Triatix-PY (50%)	PYs, 80%	95%, ITN (10.53%), LLTN (100%), 2.9
Bungoma	2.68/4.47	Actelic-OP (1.6% w/w, 15%) Skana-OP and PY (50g/90kg; 15.79%)[3]	Triatix-PY (26.32%)	Doom-PY (0.03% w/w, 15.78%)	94.74%, ITN (11.10%), LLTN (88.9%), 2.11
Busia	4/6.05	Actelic-OP (1.6% w/w, 15%) Skana-OP and PY (50g/90kg; 5%)	Triatix-PY (25%)	none	90%, ITN (61.11%), LLTN (88.89%), 3.67
